# Zero Coronary Artery Calcification: A Promising Value in Acute Chest Pain Evaluation

**DOI:** 10.7759/cureus.78365

**Published:** 2025-02-01

**Authors:** Devanshi N Damani, Chanwit Roongsritong

**Affiliations:** 1 Department of Internal Medicine, Texas Tech University Health Sciences Center El Paso, El Paso, USA; 2 Department of Vascular Medicine, Lahey Hospital and Medical Center, Burlington, USA; 3 Department of Cardiology, Texas Tech University Health Sciences Center El Paso, El Paso, USA

**Keywords:** acute coronary syndrome, cac zero, chest pain, coronary artery disease, coronary cta

## Abstract

Chest pain is the second most common reason for emergency room visits, accounting for approximately 11 million encounters annually. While benign conditions are often the underlying cause, life-threatening diagnoses such as acute coronary syndrome remain significant. Timely, accurate, and cost-effective assessment is, therefore, critical. The 2022 American Heart Association and American College of Cardiology guidelines recommend using clinical decision pathways followed by functional tests or coronary computed tomography (CT) angiography for patients with intermediate risk of acute coronary events. However, these conventional tests face challenges, including availability and complexity. Nongated, noncontrasted cardiac CT for coronary artery calcification (CAC) detection is fast and simple, and requires minimal technical expertise. Although traditionally validated in stable coronary artery disease (CAD), recent evidence supports CAC’s utility in acute settings. Notably, the absence of CAC (known as CAC Zero) demonstrates exceptional accuracy, including a ~98% negative predictive value for CAD, and predicts excellent short- and intermediate-term prognoses in patients with acute chest pain. Despite its promise, caution is advised when applying CAC Zero in younger populations, as they are more likely to have noncalcified plaques. This article reviews recent evidence on the value and limitations of CAC Zero in evaluating acute chest pain.

## Introduction and background

Acute chest pain is one of the most common presenting complaints in the United States emergency departments (EDs) [1]. Current guidelines from major United States professional organizations recommend initial risk stratification using established clinical decision pathways based on basic clinical features, electrocardiogram (ECG) findings, and initial troponin levels [2]. Patients with clear-cut acute coronary syndrome (ACS) or a nonnegligible risk (≥1%) of major adverse cardiovascular events (MACE), such as death, myocardial infarction (MI), or urgent coronary revascularization, within 30 days are typically admitted for further evaluation. Most of these patients undergo noninvasive imaging, such as stress echocardiography, myocardial perfusion imaging (MPI), or coronary computed tomography angiography (CTA). Notably, many low-risk patients also undergo further testing despite guideline recommendations to the contrary [1,2].

Some have advocated coronary CTA, a noninvasive imaging modality for evaluating atherosclerotic coronary artery disease (CAD), as a preferred diagnostic method. It demonstrates superior diagnostic accuracy compared with stress MPI, with studies reporting a sensitivity of 91%-95% and a negative predictive value (NPV) of 93%-99% for obstructive CAD (≥70% stenosis) [3-6]. However, coronary CTA has limitations, including high radiation exposure, the need for intravenous contrast, and technical expertise, which restrict its feasibility in many ED settings [3,4,6-8]. Furthermore, recent studies indicate that early coronary CTA offers no overall outcome benefits for Global Registry in Acute Coronary Events (GRACE) score-based intermediate-risk ACS patients, as it modestly reduces invasive coronary angiography rates but increases downstream testing and length of stay, negating potential cost savings [9].

Coronary artery calcification (CAC), a characteristic visualized on noncontrast CT, is a well-established marker of atherosclerosis and a predictor of cardiovascular risk in asymptomatic patients with indeterminate risk [10]. The magnitude and location of CAC correlate strongly with the anatomical extent of plaque. An absence of CAC (CAC Zero) generally indicates no significant atherosclerosis. CAC Zero is associated with excellent intermediate and long-term prognosis in this population.

CAC scoring is often performed alongside coronary CTA in patients with chest pain to assess the presence and extent of CAD. Numerous studies have established the potential add-on value of CAC score to the coronary CTA findings in managing “stable” chest pain patients. However, until recently, a significant knowledge gap existed on the role of CAC in managing patients with “acute” chest pain. CAC Zero has been consistently shown to have excellent sensitivity and NPV for obstructive CAD. CAC is cost-effective and simpler to perform than other noninvasive tests, except for the less accurate ECG stress test. An up-to-date summary of the more current findings on the value of CAC Zero in acute chest pain patients is lacking. This review summarizes recent evidence on the value of CAC Zero in acute chest pain evaluation. We discuss its potential role, limitations, and comparison with conventional testing modalities. Additionally, we propose a practical approach to incorporating this low-risk, cost-effective modality into managing acute chest pain patients.

## Review

Methods

Keywords "CAC Zero" and "Coronary CTA" were used to perform a literature search using the PubMed database. Studies published between 2005 and 2023 were included. Their results and conclusions were critically analyzed in this review.

Review

CAC as an Adjunct in Decision-Making

Currently used functional tests are not perfect. Initial studies on high-risk plaques and the significance of plaque burden relied solely on anatomical features noted on invasive coronary angiography, which were used as reference standards for significant CAD. However, it is well established that relying on anatomical findings alone is less representative of in vivo hemodynamics and has significant limitations [11]. Consequently, functional tests like single-photon emission computed tomography (SPECT) MPI and stress echocardiography began to be performed in patients with suspected CAD, supported by strong clinical evidence of their accuracy [11]. However, SPECT MPI, like other functional testing, has the limitation of detecting only flow-limiting plaques and not the total plaque burden. Thus, patients with normal stress testing may still have a significant plaque burden. Pooled, patient-level, sensitivity and specificity of 88% and 61%, respectively, for SPECT MPI with a diagnostic ratio of 15.31, have been reported from a meta-analysis of 17,901 patients by Jaarsma et al. [11]. In contrast, an alternative imaging test, coronary CTA, is noted to have better results. As seen in the Prospective Multicenter Imaging Study for Evaluation of Chest Pain (PROMISE) trial, a negative CTA has been shown to have a lower three-year CAD event rate (0.9%) compared with negative stress testing (2.1%). In a prespecified analysis of the PROMISE trial, patients with diabetes who underwent coronary CTA had a lower cardiovascular death or MI than those randomized to stress testing (hazard ratio, HR: 0.38; 95% confidence interval: 0.18-0.79; p = 0.01) [12].

A European multicenter study using invasive functional assessment in addition to anatomically defined diameter stenosis as indicators for significant CAD has reported a sensitivity of 74% and specificity of 73% for SPECT MPI [3]. More recently, Takx et al. published their meta-analysis using invasive coronary angiography with fractional flow reserve as a reference standard comparing the performance of various MPI modalities. They reported poorer performance for SPECT than previously documented, with a vessel-level sensitivity and specificity of 61% and 84% (area under the curve, AUC: 0.83) and a patient-level sensitivity and specificity of 74% and 79% (AUC: 0.82), respectively [13]. Sensitivity and specificity for stress echocardiography were 69% and 84%, respectively, with an AUC of 0.83 at the patient level. The authors concluded that MPI with magnetic resonance imaging, positron emission tomography, or computed tomography may be better suited than SPECT or echocardiography alone [13]. Although better performance has been reported in another meta-analysis, including slightly larger numbers of studies, the accuracy of SPECT in detecting significant CAD was still in the moderate range [12,14,15].

In a study with 2,050 subjects, the CAC score added to the clinical model pretest probability of CAD more accurately predicted negative MPI than clinical probability alone. The pretest probability of the aforementioned study based on the American College of Cardiology/American Heart Association model was 27%, with an NPV of 98% [15]. Furthermore, the role of CAC in guiding more selective use of subsequent testing (coronary CTA or functional testing) in symptomatic stable chest pain patients has specifically been evaluated in randomized trials. In the CRESCENT trial, 350 subjects with stable chest pain were randomized to CAC scanning versus functional testing. Only patients with detectable CAC or high pretest risk underwent follow-up coronary CTA. At 1.2 years, the CAC arm was associated with a reduction in CV events (96.7% vs. 89.8% event-free survival) compared with those who underwent exercise testing alone (p = 0.011) [16].

In the CRESCENT-II trial, comprehensive cardiac CT protocol guided by CAC was compared with functional testing in 268 patients with a mean age of 58 years. In the tiered cardiac CT protocol, coronary CTA was performed only if coronary calcification was detected. At six months, the event rates were similar between the two groups. However, the rate of invasive angiograms without the European Society of Cardiology Class I indication for revascularization was lower in the CT group (1.5 % vs. 7.2%, p = 0.035) [17].

These trials support the fact that a combination of clinical probability and anatomical and functional features can optimize the use of appropriate testing. They also highlight the potential value of incorporating the CAC test into the management protocol of stable chest pain patients [16,17].

The Role of CAC Zero and Coronary CTA in Chest Pain Evaluation

With advancements in coronary CTA technology, atherosclerotic plaque constituents, atherosclerotic plaque constituents, and high-risk plaque characteristics can now be identified with greater accuracy. Positive remodeling, a thin fibrous cap, a large necrotic core, low attenuation plaque, and microcalcifications are considered to be associated with plaque vulnerability. In a post hoc analysis of the Scottish Computed Tomography of the Heart (SCOT-HEART) trial, these features provide insights into the high risk of subsequent ACS even without flow-limiting stenosis [18]. This multicenter randomized controlled trial investigated coronary CTA in outpatients with suspected angina pectoris due to CAD. Obstructive CAD was defined as >70% stenosis in one or more of the major epicardial coronary vessels or >50% stenosis of the left main. The mean age of the study participants was 58 years. Of 4,146 subjects randomized, 1,778 underwent coronary CTA, of which 37% had normal coronary artery and 26% had obstructive CAD. However, the median CAC score for subjects with adverse plaque was significantly higher than those without adverse plaques (281 vs. 0, p < 0.001) and were more likely to have prior CAD. The interquartile range of CAC scores in those with adverse plaque was 89-775. In the multivariate analysis, the predictive value of the presence of adverse plaque on MACE was dependent on the CAC score [19]. Furthermore, the same group of investigators reported an excellent prognosis for up to one year in subjects with CAC Zero regardless of the presence of adverse plaque or obstructive CAD (2/689 event rate or 0.003%) [18,19].

Osborne-Grinter et al. recently reported their findings of more detailed qualitative and quantitative analyses on the relationship between CAC and adverse plaque from the SCOT-HEART trial dataset. Of 1,769 patients with stable chest pain, 642 (36%) had CAC Zero. In this subgroup, 84% of them had normal coronary arteries, 14% had nonobstructive CAD, and 2% had obstructive CAD on coronary CTA. Adverse plaque features were present in 2% of CAC Zero patients. Notably, the highest risk plaque feature, defined as a low-attenuation plaque burden >4%, was found in 13% of patients with adverse plaques, corresponding to two in 642 or a 0.3% rate of high-risk plaques in CAC Zero patients [20]. Interestingly, the authors concluded that while a CAC score of zero is associated with a good prognosis, it does not entirely rule out obstructive CAD, nonobstructive plaque, or adverse plaque phenotypes, including low-attenuation plaque.

Similarly, other studies have supported using CAC Zero as a highly sensitive measure with a strong NPV for CAD. In the Coronary CT Angiography Evaluation for Clinical Outcomes: An International Multicenter study, 10,037 patients with stable chest pain without known CAD who underwent concomitant CAC and coronary CTA were identified from the registry. The mean age was 57 years, and 51% had CAC Zero. Among the patients with CAC Zero, obstructive CAD, defined as ≥50% stenosis, was found in 3.5%, whereas 1.4% had ≥70% stenosis. The sensitivity and NPV of CAC Zero for ≥50% stenosis were 89% and 96%, respectively. During a median follow-up of 2.1 years, those with obstructive CAD had a higher composite endpoint (3.9% vs. 0.8%), but there was no difference in mortality among patients with CAC Zero irrespective of obstructive CAD [21].

In a prospective study involving 1,753 patients with stable chest pain undergoing CAC scoring with or without coronary CTA, Wang et al. reported a CAC Zero score in 915 subjects (52.2%). Of the 751 patients with CAC Zero who underwent coronary CTA, 89.7% had normal coronary arteries, and only 1.9% had obstructive CAD. The NPV of a CAC Zero score for obstructive CAD was 98.1%. Over a median follow-up of 2.2 years, the absolute annualized rates of MACE for CAC Zero were very low at 1.9 per 1,000 person-years [22]. In another observational study of 668 patients with chest pain syndrome (61% chronic and 39% with acute presentation), Rubinshtein et al. reported a 7% incidence of angiographically confirmed obstructive CAD in 125 subjects with a CAC score of zero. Although 76% of this subgroup subsequently required myocardial revascularization, no death or MI was reported [23]. Agha et al., in their study on patients with stable chest pain, demonstrated that CAC Zero predicted a reassuringly low annual rate of MACE and mortality at 0.5% and 0.3%, respectively [24].

Taken together, the prevalence of obstructive CAD in patients with CAC Zero appears to be very low. CAC Zero has excellent NPV for obstructive CAD (96%-98%) and offers reassuring prognostic information for subsequent cardiovascular events up to one to two years in stable chest pain patients.

The Value of CAC in Patients With Acute Chest Pain

As mentioned, the current guidelines do not recommend the CAC test as a risk stratification tool for acute chest pain. This is based on the well-established pathological fact that, in its early stages, atherosclerotic plaques are not calcified. Vulnerable plaques are often lipid-rich with a thin cap, which is not detectable by the CAC test. CAC Zero varies by age among patients enrolled in studies evaluating the potential usefulness of coronary CTA for acute chest pain. Additionally, obstructive CAD, defined as ≥50% diameter stenosis, has been observed in coronary CTA in patients with acute chest pain despite the absence of CAC. Rates as high as 38% have been reported in relatively small retrospective case series [25]. In a study of 146 patients admitted to the coronary care unit for evaluation of acute chest pain, Correia et al. reported that 35% had CAC Zero. The overall prevalence of obstructive CAD (defined as ≥70% stenosis by invasive coronary angiography) was 41%. In those with a pretest probability of <50%, CAC Zero had an NPV of 95% [26].

Recently, registry data involving a substantially larger patient cohort has provided additional insights. Grandhi et al. analyzed data from their Chest Pain Registry data of 5,192 patients presenting to the ED with acute chest pain classified as low-to-intermediate risk patients based on thrombolysis in myocardial infarction (TIMI) risk score with normal or nondiagnostic ECG and negative serum troponin [27]. The prevalence of CAC Zero was 56%. Among those with CAC Zero, 4.6% had CAD, but only 0.7% fell into the obstructive range. The sensitivity and NPV for obstructive CAD for CAC Zero were 96.2% and 99.3%, respectively. More importantly, only 0.4% of patients with CAC Zero required coronary revascularization [27].

Furthermore, Agha et al. recently published a very large meta-analysis of over 92,000 patients with low-to-intermediate risk for CAD undergoing coronary CTA. Among 12,376 subjects presenting with acute chest pain, CAC Zero was observed in 58%. In this particular subgroup, the prevalence of nonobstructive CAD was 9%, and only 4% had obstructive CAD. The NPV of CAC Zero for an obstructive CAD was remarkably high at 98%. Notably, the short-term rates of MACE (30 days for most studies), revascularization, and ACS for subjects with CAC Zero were 0.8%, 0%, and 0.3%, respectively [24].

CAC Zero and Short-Term Outcome in Acute Chest Pain

Any novel test or therapy must be clinically deployed with sufficient evidence for reliability, reproducibility, and safety to provide high-quality patient care. Several investigators have studied the association of CAC Zero in patients with acute chest pain and their clinical outcomes over time. Some of the studies are summarized below.

In a pooled analysis of patients presenting to the ED with chest pain classified as low-to-intermediate risk based on several traditional risk stratifications (Framingham, TIMI, Diamond Forrester), a group of investigators from Johns Hopkins reported a 99.4% NPV of CAC zero for coronary heart disease events, on an average follow-up of 21 months [25].

In another study of patients seen in a rapid-access chest pain clinic of three hospitals in the United Kingdom, 300 consecutive subjects were followed for a mean of 17 months. None of the subjects with CAC Zero (44% of the total population) experienced cardiac events [28].

Chaikriangkrai et al. analyzed the value of CAC in 3,556 patients without CAD from three studies. The pooled prevalence of CAC Zero was 60%. Over a median follow-up of 10.5 months, the pooled event rates for CAC Zero without ischemic ECG changes or positive biomarkers were very low at 0.8% per year, compared with 14.6% per year for those with CAC greater than zero [29].

A more recent report on patients with acute chest pain published by Bittner et al. examined the outcomes in 795 patients with CAC Zero. The rate of subsequent acute coronary events was extremely low at 0.5% (one MI and three unstable angina pectoris). The results showed that 39 out of 795 patients were deemed indeterminate. Of these, 31 underwent further testing during the index admission. Eighty-three patients had 1%-49% stenosis, and 13 patients had greater than 50% stenosis. The cost of downstream testing per ACS is $464,399 [30].

In the aforementioned meta-analysis by Agha et al., the incidence of short-term MACE, revascularization, and ACS for subjects with CAC Zero was extremely low. As expected in patients with stable chest pain, CAC Zero predicted a reassuringly low annual rate of MACE and mortality at 0.5% and 0.3%, respectively [24].

In the Rapid Assessment of Potential Ischemic Heart Disease with Coronary Computed Tomography Angiography study of acute chest pain, the total plaque burden, rather than the presence of obstructive CAD, predicts subsequent future MACE [9,31].

Table 1 summarizes the studies and their primary results. The studies show that CAC Zero offers promising prospects for testing patients with acute and stable chest pain.

**Table 1:** Studies on the diagnostic accuracy and clinical utility of CAC zero in patients with stable and acute chest pain ACS: acute coronary syndrome; AU: Agatston units; AUC: area under the curve; CAC: coronary artery calcium; CAD: coronary artery disease; CCTA: coronary computed tomography angiography; CT: computed tomography; CV: cardiovascular; HR: hazard ratio; LR-: negative likelihood ratio; LR+: positive likelihood ratio; MACE: major adverse cardiovascular event; MI: myocardial infarction; MPI: myocardial perfusion imaging; NNT: number needed to treat; NPV: negative predictive value; OR: odds ratio; PET: positron emission tomography; PPV: positive predictive value; RR: risk ratio

Study	Aim	Number of subjects	Method	Primary result
Clerc et al. [15]	CAC vs. pretest probability vs. combination to predict perfusion defects in PET in suspected CAD	2,050	Prospective, observational, cross sectional	CAC and combination have better discriminatory power than pretest probability and are a stronger predictor of myocardial PET findings (23%-37% vs. 2%-7%)
Lubbers et al. [16]	Tiered cardiac CT (CAC à CCTA) as an alternative to functional testing in patients with stable CAD	350	Prospective, randomized	CT group had higher event-free survival (96.7% vs. 89.8%, p = 0.011) and were diagnosed with CAD sooner with less downstream testing (25% vs. 53%; p < 0.0001) at the end of 1.2 years
Lubbers et al. [17]	Tiered cardiac CT (CAC à CCTA à CT MPI) as an alternative to functional testing in patients with stable CAD	268	Multicenter, prospective, randomized	Lower rate of invasive coronary angiograms in the CT group compared with functional testing at six months (1.5% vs. 7.2%, p = 0.035)
Williams et al. [18]	Adverse coronary plaque characteristics and their prognostic implications in patients with suspected CAD	1,769	Post hoc, multicenter, randomized controlled trial	For CAC <100 AU, patients with adverse plaque were at an increased risk of CAD death or nonfatal MI compared with patients without adverse plaque (HR: 3.38; p = 0.03). For CAC >100 AU, no significant difference in patients with or without adverse plaque was noted
Williams et al. [19]	Association between low-attenuation plaque burden, CV risk score, obstructive CAD, or CAC to future risk of fatal or nonfatal MI	1,769	Post hoc, multicenter, randomized controlled trial	Low-attenuation plaque burden is the strongest predictor of MI (HR: 1.60; p = 0.014) and patients with >4% burden are five times more likely to have an MI (HR: 4.65, p < 0.001)
Villines et al. [21]	Prevalence of CAD and clinical outcomes in symptomatic patients who underwent CCTA and had CAC zero	10,037	Multicenter, prospective, observational	At 2.1-year follow-up in patients with CAC zero, there was no difference in all-cause mortality in obstructive/nonobstructive CAD. CAC zero added no incremental prognostic information compared with CCTA (p = 0.84)
Agha et al. [24]	CAC to rule out obstructive CAD in low-to-intermediate risk patients with acute and stable chest pain	92,279 (stable: 79,903; acute: 12,376)	Systematic review and meta-analysis	CAC zero was associated with a low prevalence of nonobstructive and obstructive CAD and a low annualized risk of MACE
Wang et al. [22]	Study the prevalence of noncalcified CAD in symptomatic patients with CAC zero and its prognostic implications	915	Prospective, observational	CAC zero had an NPV of 98.1% for stenosis ≥50%. No difference in the all-cause mortality (p = 0.27) and MACE (p = 0.19) in patients with or without CAC zero was noted
Rubinshtein et al. [23]	Study the extent of CAD in symptomatic patients with zero or low CAC	231	Prospective, observational	All patients with CAC zero had single-vessel disease. Zero or low CAC does not completely exclude obstructive CAD in symptomatic patients
Grandhi et al. [27]	Diagnostic accuracy of CAC to rule out obstructive CAD in low-to-intermediate risk patients presenting with acute chest pain	5,192	Prospective, observational	For the diagnosis of obstructive CAD, CAC had a sensitivity of 96.2%, specificity of 62.4%, PPV of 22.4%, and NPV of 99.3%. NPV for patients needing revascularization are 99.6% and NNT are 264
Correia et al. [26]	Gatekeeping role of CAC zero in patients with acute chest pain with a high pretest probability	146	Prospective, observational	CAC zero was independently associated with no CAD (OR: 0.12, p < 0.001). CAC scoring enhanced the AUC from 0.76 to 0.83 (p = 0.006)
Yerramasu et al. [28]	Can CAC zero safely exclude obstructive CAD in patients with low-risk and stable chest pain? Determine if 400 AU is an appropriate threshold for the usefulness of CCTA	300	Prospective study	CAC zero with low pretest probability: none had CAD or an MACE. CAC ≥1: sensitivity = 96%, specificity = 53%, NPV = 32%, and PPV = 98%. CAC >400 AU: high prevalence of obstructive CAD
Chaikriangkrai et al. [29]	Accuracy and prognostic value of CAC zero to identify patients with low risk of CV events	3,556	Systematic review and meta-analysis	Pooled analysis had a sensitivity of 96%, a specificity of 60%, an LR+ of 2.36, and an LR- of 0.07. CAC zero had a significantly lower risk of MACE (RR: 0.06) and MI/death (0.19) compared with patients with CAC >0
Bittner et al. [30]	Determine if CAC affects the efficiency, cost, and diagnostic yield of CCTA in patients with suspected ACS	1,234	Multicenter observational cohort analysis of randomized controlled trials	Excellent diagnostic yield with high CAC. With increasing CAC, downstream testing and cost increase but in an appropriate fashion
Mortensen et al. [32]	Diagnostic value of CAC zero for obstructive CAD and its implications	23,759 (CAC zero: 12,771)	Cohort study	CAC zero’s ability to rule out obstructive CAD depended on the patient’s age and the value added was smaller in younger patients
Osborne-Grinter et al. [20]	Study the association of CAC with plaque subtypes	1,769	Post hoc, prospective open-label, parallel-group, multicenter trial	Extremes of the CAC score spectrum can miss at-risk patients

Current Status of ACS in the United States and Developed World

Studies on the triage and work-up of acute chest pain using current decision pathways have shown varying rates of ACS and MACE. In the Rule Out Myocardial Infarction/Ischemia Using Computer-Assisted Tomography II trial that enrolled 1,000 patients between 40 and 74 years of age with acute chest pain suspected of ACS but without ischemic ECG changes or positive initial troponin, the rate of ACS was 8% [7]. In comparison, Stopyra et al. reported a 10.2% incidence of 30-day MACE in 5,799 patients presenting to ED with symptoms suggestive of ACS [33]. Conversely, Mark et al., in a very large prospective cohort study of 13,192 adult patients with acute chest pain presenting to the ED at 13 medical centers within an integrated healthcare system, reported that the incidence of 60-day MACE is at 3.7% [34].

Risk stratification for CAD is essential in the ED for all patients with chest pain. In addition to clinical features, the presenting ECG, and standard troponin tests, more accurate tests have been sought to reduce the rate of missed ACS cases. Incorporating coronary CTA in the initial evaluation of patients has improved the efficiency of clinical decision-making and reduced the length of stay and hospitalizations. However, it has also increased radiation exposure without any change in 28-day MACE or overall care costs [7].

In contrast, the availability of high-sensitivity troponin has proven to be highly valuable, significantly reducing the incidence of true ACS after an initially negative biomarker. As demonstrated in the GRACE and TIMI trials, patients with a negative biomarker are generally at a lower risk for ACS [35,36]. Reichlin et al. showed that using high-sensitivity cardiac troponin increased the detection of acute MI by 4%. They found that among all of their patients with ACS, about 11% were classified as having unstable angina [37].

Some experts have argued that troponin-negative unstable angina would be better categorized as a subtype of severe stable angina [38]. However, others caution against this reclassification, especially in higher risk groups such as older adults, patients with heart failure, or low blood pressure, to avoid missing ACS diagnoses [37].

Limitations in the use of CAC and coronary CTA

*Distinction Between Acute and Stable Chest Pain and Consistency of Definition Among Various Trials*​​​​​​

Current guidelines categorize patients with chest pain into two groups, acute and stable, to stratify risk and make decisions about further management. The CAC score is a class IIa recommendation for the initial evaluation of patients with stable chest pain, but the guidelines do not provide any specific recommendation on CAC in acute chest pain.

In the current guidelines, acute chest pain is defined as substernal chest discomfort precipitated by exertion or emotional stress and relieved by rest or nitroglycerin. However, data published in systematic reviews and meta-analyses on risk scores use phrases such as “chest pain presenting to the ED” or “chest pain in the ED” as inclusion criteria. These terms are vague and do not clearly distinguish between acute and stable chest pain. It is likely that some, if not most, studies enrolled both groups of patients as defined by the current guidelines.

For instance, in the meta-analysis by Agha et al., studies enrolling subjects with acute chest pain and/or being evaluated in the ED with chest pain were considered acute chest pain and analyzed together [24,36,39]. In another study by Jia et al., on the evaluation of intracoronary imaging of patients with ACS using Massachusetts General Hospital (MGH) Optical Coherence Tomography (OCT) Registry data, unstable angina was defined as newly developed or accelerating chest symptoms on exertion or rest angina within two weeks [40].

Therefore, the role of CAC likely should not be strictly confined to either subgroup. Furthermore, in real-world scenarios, a significant number of patients presenting to the emergency room for evaluation of chest pain do not meet the guideline definition of chest pain. Some could be arguably classified as stable chest pain. Hence, CAC could be a valuable tool in evaluating appropriate patients presenting to ED with chest pain.

Age and the Value of CAC Zero

Age plays a critical role in calcifying the atherosclerotic plaque and thus affects the CAC score. The diagnostic value of CAC Zero to rule out obstructive CAD depends on the patient’s age in cases of stable chest pain [32]. Most prior studies on the value of coronary CTA and CAC score in symptomatic chest pain were conducted in the middle-aged population, with the median age in the high 40s or 50s. Some authors argue that CAC is an unreliable diagnostic tool in younger symptomatic patients (<40 years of age) [41].

In a recently published study by Mortensen et al. from the Western Denmark Heart Registry, which included 23,759 patients who underwent coronary CTA for suspected CAD symptoms, the mean age was 58 years. Among these patients, 54% had CAC Zero, and this CAC Zero was especially common (93%) in those younger than 40 years. Obstructive CAD was very infrequent (3%) in patients less than 40 years, but 58% of patients younger than 40 years with obstructive CAD had CAC Zero [32].

Additionally, Nagasawa et al. recently reported their findings in a retrospective study of 436 patients who underwent preintervention OCT. Multivariate analysis revealed that thin-cap fibroatheroma and ST-segment elevation MI were independently associated with plaque rupture in younger patients (less than 60 years old). Conversely, unstable angina and non-ST-segment elevation MI showed a lower incidence of thin-cap fibroatheroma and were independently associated with calcified nodules [42]. Thus, the patient’s age is important when interpreting CAC scores or culprit lesion characteristics.

A Practical Approach for CAC Zero in Acute Chest Pain

Considering all the above observations, a practical approach for applying CAC Zero in clinical practice would be to utilize the test in more selected populations with acute chest pain. The pretest probability of CAD in men under 40 years and women under 50 years with stable chest pain is less than 15%, and further testing is generally not recommended in the current guidelines. Given the possibility of noncalcified but flow-limiting and/or unstable plaque in younger subjects, though the prevalence is low, the subgroup of patients with acute chest pain where CAC Zero would be more optimally utilized as an initial diagnostic tool we believe are men 40 years old or older (greater than 45-50 years for women), with no known CAD, negative initial biomarker, no evidence of ischemia on ECG, and a low-to-intermediate risk clinical risk.

## Conclusions

Accurate and timely diagnosis of cardiac chest pain remains a critical focus, with substantial efforts directed at minimizing missed ACS. Recent studies on functional testing have shown that coronary CTA has a better NPV than SPECT MPI and stress echo and hence a more effective tool to rule out obstructive CAD. The potential of CAC scores in patients with significant obstructive CAD has been increasingly recognized. CAC scores consistently exhibit high sensitivity and NPV, making them valuable in clinical decision-making. Incorporating CAC Zero in the clinical pretest probability model can further enhance the prediction of CAD. CAC scoring, as an initial diagnostic tool, can lead to more efficient triage, reduced healthcare costs, and improved patient outcomes in real-world settings.

However, there are limitations in the generalizability of the CAC score as the definitions of chest pain in the various studies were not uniform. Another consideration is its cautious application in younger patients who are more likely to have uncalcified plaques. Hence, the pretest probability and clinical scenario are essential when using the CAC score in triage. When used in an appropriate clinical setting, the CAC Zero correlated with excellent short- and intermediate-term prognoses in patients with acute chest pain.

Further studies on long-term outcomes of patients triaged based on the CAC score with subgroup analysis can add to the knowledge base. Novel approaches exploring the optimization of the CAC score in clinical practice could guide future practice.
